# Supplementary material for: Engineering of acyl ligase domain in non-ribosomal peptide synthetases to change fatty acid moieties of lipopeptides
Source: Commun Chem. 2025 Jan 21;8:17. doi: 10.1038/s42004-024-01379-w (PMC11751314; doi:10.1038/s42004-024-01379-w)
Supplement: Supplementary file 2 — Supplementary Information [file 42004_2024_1379_MOESM2_ESM.pdf]

Supplementary Information

Engineering of acyl ligase domain in non-ribosomal peptide synthetases to change fatty acid moieties of lipopeptides

Rina Aoki[1], Eri Kumagawa[2], Kazuaki Kamata[2], Hideo Ago[3], Naoki Sakai[3, 6], Tomohisa Hasunuma[4, 5], Naoaki Taoka[1], Yukari Ohta[2, 7], Shingo Kobayashi[1]

[1] Agri-Bio Research Center, KANEKA CORPORATION, 1-8, Miyamae-cho, Takasago-cho, Takasago, Hyogo 676-8688, Japan,

[2] Gunma University Center for Food Science and Wellness, Gunma University, 4-2 Aramaki, Maebashi, Gunma 371-8510, Japan

[3] RIKEN SPring-8 Center, 1-1-1 Kouto, Sayo-cho, Sayo-gun, Hyogo 679-5148, Japan

[4] Graduate School of Science, Technology, and Innovation, Kobe University, 1-1 Rokkodai, Nada, Kobe 657-8501, Japan

[5] Engineering Biology Research Center, Kobe University, 1-1 Rokkodai, Nada, Kobe 657-8501, Japan

[6] Present address: Japan Synchrotron Radiation Research Institute, 1-1-1 Kouto, Sayo-cho, Sayo-gun, Hyogo 679-5198, Japan

[7] Present address: Laboratory of Food Microbiology, Department of Life and Food Sciences, School of Life and Environmental Sciences, Azabu University, 1-17-71 Fuchinobe, Chuo-ku, Sagamihara, Kanagawa, 252-5201, Japan

Corresponding author: Shingo Kobayashi (Shingo.Kobayashi@kaneka.co.jp)

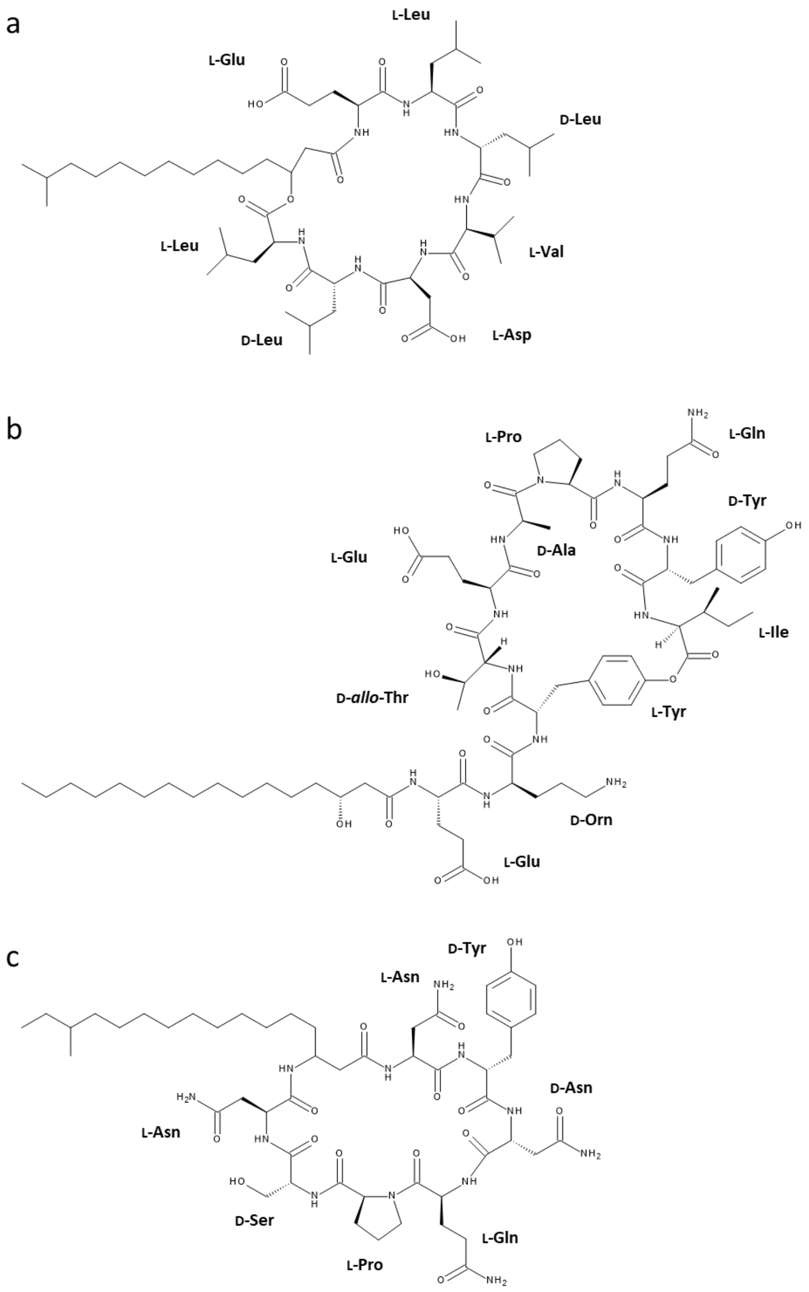

Supplementary Figure 1. **Structure of CLPs. a)** A surfactin homologue with an *iso*-C15 fatty acid moiety. **b)** A plipastatin A homologue with a *n*-C16 fatty acid moiety. **c)** A mycosubtilin homologue with an *anteiso*-C17 fatty acid moiety.

```
ItuAL  1    MYTSQFQTLVDVIRERSSISDRGIRFIESDKNETVVSYRQLFEEAQGYLGYLQHLGIKPK  60
            ||||||||||||||.||.||||||||||||| || |||||||.||||.||||||.||.||
MycAL  1    MYTSQFQTLVDVIRNRSNISDRGIRFIESDKIETFVSYRQLFDEAQGFLGYLQHIGIQPK  60

ItuAL  61   QEIVFQIQENKSFVVAFWACILGGMIPVPVSIGEDDDHKLKVWRIWNILNHPFLIASEKV  120
            ||||||||||||||||||||.||||||||||||||.||||||||||||||.|||.|||.|
MycAL  61   QEIVFQIQENKSFVVAFWACLLGGMIPVPVSIGEDNDHKLKVWRIWNILNNPFLLASETV  120

ItuAL  121  LDKIKKYAAEHDLQDFHHQLNEKSDIIQDQTYDYPASFYEPDADELAFIQFSSGSTGDPK  180
            |||.||.||.|||||||||| ||||||||..||.||| |||.||||||||||||||||||
MycAL  121  LDKMKKFAADHDLQDFHHQLIEKSDIIQDRIYDHPASQYEPEADELAFIQFSSGSTGDPK  180

ItuAL  181  GVMLTHHNLIHNTCAIGTALAIHSKDSFLSWMPLTHDMGLIACHLVPFITGINQNLMPTE  240
            |||||||||||||||| .||||. ||..||||||||||||||||||| ..||||||||||
MycAL  181  GVMLTHHNLIHNTCAIRNALAIDLKDTLLSWMPLTHDMGLIACHLVPALAGINQNLMPTE  240

ItuAL  241  LFIRRPILWMKKAHEHKASILSSPNFGYNYFLKFLKNE--PDWDLSHIKVIANGAEPILP  298
            ||||||||||||||||||||||||||||||||||||..   |||||||.|||||||||||
MycAL  241  LFIRRPILWMKKAHEHKASILSSPNFGYNYFLKFLKDNKSYDWDLSHIRVIANGAEPILP  300

ItuAL  299  ELCDEFLKRCAAFNLKRSAILNVYGLAEASVGAAFSKIGKEFVPVYLHRDYLNLGERAVN  358
            |||||||.||||||.||||||||||||||||||.||.||. |||||||||.||||||||.
MycAL  301  ELCDEFLTRCAAFNMKRSAILNVYGLAEASVGATFSNIGERFVPVYLHRDHLNLGERAVE  360

ItuAL  359  VSKEDQNCASFVEVGQPIDYCQLRISDETNERVEDGIIGHIQIKGDNVTQGYYNNPESTE  418
            |||||||||||||||.||||||.||..|.|| .|||.||||||||.|||||||||||||.
MycAL  361  VSKEDQNCASFVEVGKPIDYCQIRICNEANEGLEDGFIGHIQIKGENVTQGYYNNPESTN  420

ItuAL  419  KVLTSDGWVKTGDLGFISESGNLVVTGREKDIIFVNGKNIYPHDIERVAIEMEEVDLGRV  478
            ..||.||||||||||||. .|||||||||||||||||||.|||||||||||.|..|||||
MycAL  421  RALTPDGWVKTGDLGFIR-KGNLVVTGREKDIIFVNGKNVYPHDIERVAIELEDIDLGRV  479

ItuAL  479  AACGVYDQKTQSGEIVLFVVYKKSPEKFAPLVKEIKKHLYKRGGWSIKEVLPIRKLPKTT  538
            ||||||||.|.| |||||.|||||.|.||||||.||||||.||||||||.||||||||||
MycAL  480  AACGVYDQETRSREIVLFAVYKKSAEQFAPLVKDIKKHLYQRGGWSIKEILPIRKLPKTT  539

ItuAL  539  SGKVKRYELARQYEAGNFSTESAAINECLESSPETSGQTPIHEIET  584
            |||||||||| |||.|.|. ||. |.| ||. .... |||||||||
MycAL  540  SGKVKRYELAEQYESGKFALESTKIKEFLEGHSTEPVQTPIHEIET  585
```

Supplementary Figure 2. Amino acid sequence alignment of ItuAL derived from *Bacillus subtilis* RB14 and MycAL derived from *B. subtilis* ATCC6633. Vertical lines connecting the top and bottom amino acid residues indicate that the two amino acids are identical. Points between the top and bottom amino acids indicate that the two amino acids are similar. The identity and similarity of the two sequences are 84 and 96%, respectively. Amino acid residues mediating the specificity of fatty acids as substrates are shown in bold. For generating chimaeric acyl ligase (AL) domains, we divided them into five regions. Regions 1, 2, 3, 4, and 5 are shown in pink, yellow, green, blue, and purple backgrounds, respectively.

```
ItuACP  1   ELLSIFSEVLNGKKVHLADSYFDMGANSLQLSQIAERIEQKFGRELAVSDLFTYPSITDL  60
            .||||||||..|||.||.| ||||||.|||||||||||||||| ||.|.||||||||.||
MycACP  1   ALLSIFSEVMDGKKIHLNDHYFDMGATSLQLSQIAERIEQKFGCELTVADLFTYPSIADL  60

ItuACP  61  AAYLSESRAEIKQDAAAKPSHVTPKD  86
            ||.| |...||||...||||. ..||
MycACP  61  AAFLVENHSEIKQTDTAKPSRSSSKD  86
```

Supplementary Figure 3. Amino acid sequence alignment of ItuACP derived from *Bacillus subtilis* RB14 and MycACP derived from *B. subtilis* ATCC6633. Vertical lines connecting the top and bottom amino acid residues indicate that the two amino acids are identical. Points between the top and bottom amino acids indicate that the two amino acids are similar. The identity and similarity of the two sequences are 74 and 95%, respectively.

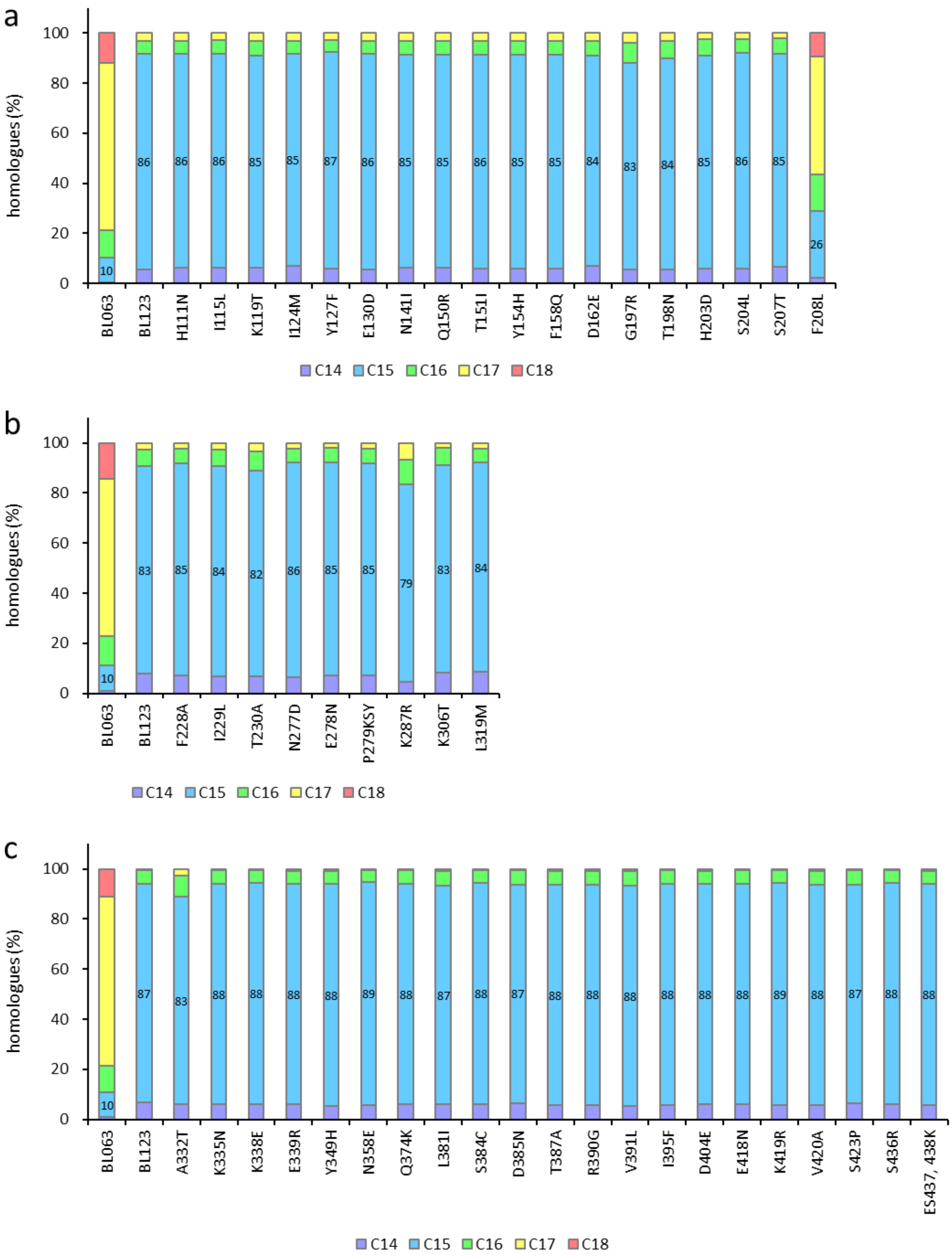

Supplementary Figure 4. **Relative abundance of mycosubtilin homologues produced by mutant strains containing ItuAL with an amino acid substitution mutation. a)** region 2, **b)** region 3, **c)** region 4.

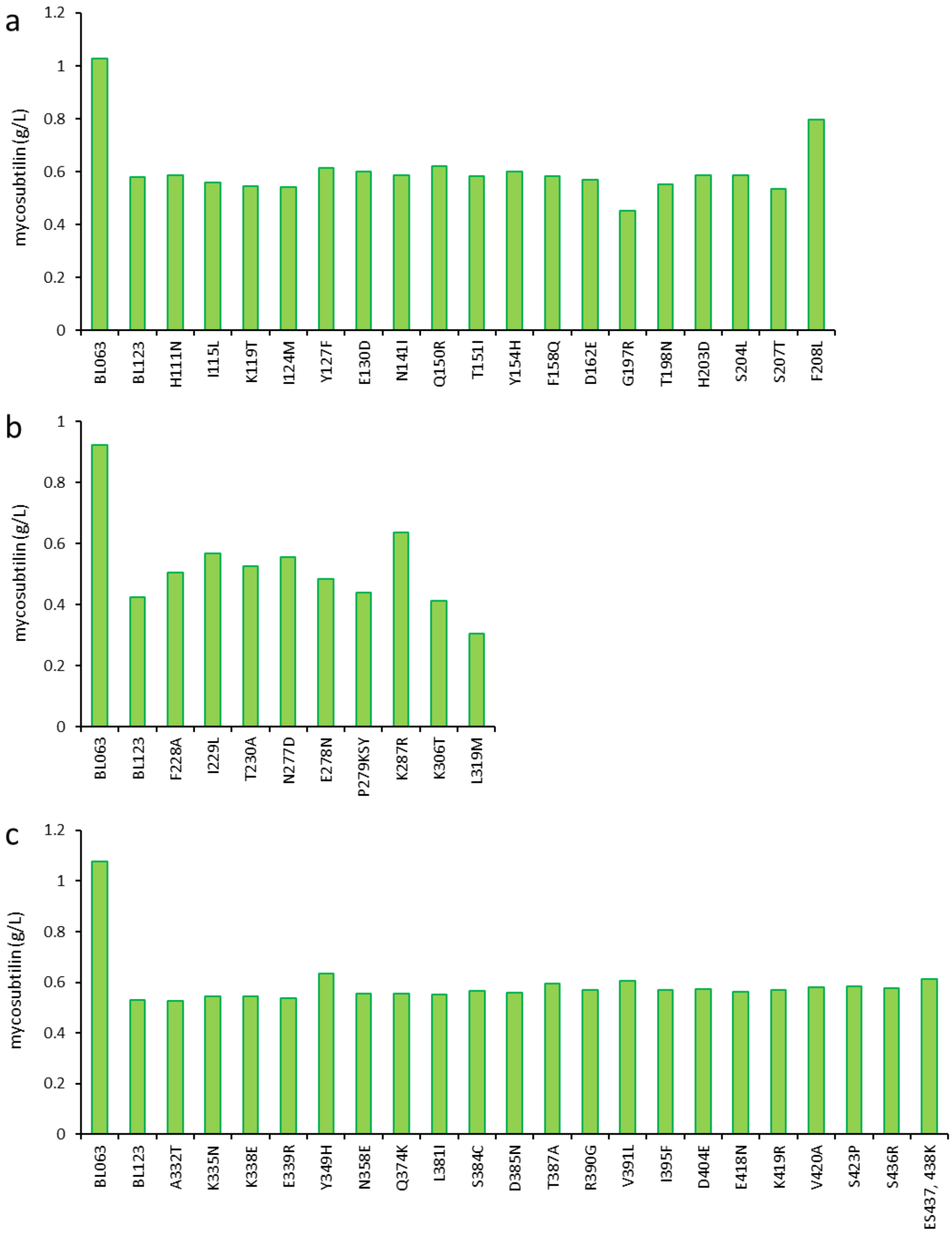

Supplementary Figure 5. **Productivity of mycosubtilin produced by mutant strains containing ItuAL with an amino acid substitution mutation. a)** region 2, **b)** region 3, **c)** region 4.

```
iturin A          1 MYTSQFQTLVDVIRERSSISDRGIRFIESDKNETVVSYRQLFEEAQGYLGYLQHLGIKPK 60
bacillomycin L    1 MYTSQFQTLVDVIRERSSISDRGIRFIESDKNETVVSYRQLFEEAQGYLGYLQHLGIKPK 60
bacillomycin D    1 MYTSQFQTLVDVIRERSNISDRGIRFIESDKNETVVSYRQLFEEAQGFLGYLQHIGIQPK 60
bacillomycin F    1 MYTSQFQTLVDVIRERSNISYRGIRFIESDKKETVVSYRQLFDEAQGFLGYLQHIGIQPK 60
mycosubtilin      1 MYTSQFQTLVDVIRNRSNISDRGIRFIESDKIETFVSYRQLFDEAQGFLGYLQHIGIQPK 60
mojavensin        1 MFTNQFQTLVDVIRERSNQTDHGIRFIESDKTEKFISYRQLFEEAQGFLGYLQHIGIQPK 60
                    *.*.**********.**.....********* *...******.****.******.**.**

iturin A         61 QEIVFQIQENKSFVVAFWACILGGMIPVPVSIGEDDDHKLKVWRIWNILNHPFLIASEKV 120
bacillomycin L   61 QEIVFQIQENKSFVVAFWACILGGMIPVPVSIGEDDDHKLKVWRIWNILNHPFLIASEKV 120
bacillomycin D   61 QEIVFQIQENKSFVVAFWACLLGGMIPVPVSIGEDDDHKLKVWRIWNILNHPFLVASEKV 120
bacillomycin F   61 QEIVFQIQENKSFVVAFWACLLGGMIPVPVSIGEDDDHKLKVWRIWNILNHPYLIASEKV 120
mycosubtilin     61 QEIVFQIQENKSFVVAFWACLLGGMIPVPVSIGEDNDHKLKVWRIWNILNNPFLLASETV 120
mojavensin       61 QEVVFQIQENKSFVVAFWACLLGGMIPVPVSIGEDDDHKLKVWRIWNILNNPFLIASEKA 120
                    **.*****************.**************.**************.*.*.***..

iturin A        121 LDKIKKYAAEHDLQDFHHQLNEKSDIIQDQTYDYPASFYEPDADELAFIQFSSGSTGDPK 180
bacillomycin L  121 LDKIKKYAAEHDLQDFHHQLNEKSDIIQDQTYDYPASFYEPDADELAFIQFSSGSTGDPK 180
bacillomycin D  121 LDKIKKYAAEHDLQDFHHQLNEKSDIIQDQTYDYPASFYEPEADELAFIQFSSGSTGDPK 180
bacillomycin F  121 LDKIKKYAAEHDLQDFHHQLNEKSDIIQDQTYDYPASIYEPDADELAFIQFSSGSTGHPK 180
mycosubtilin    121 LDKMKKFAADHDLQDFHHQLIEKSDIIQDRIYDHPASQYEPEADELAFIQFSSGSTGDPK 180
mojavensin      121 LEKIKKYAAEHDMQDFHHQLSEKSDIIQDRIYNQPASLYEPGADELAFIQFSSGSTGNPK 180
                    *.*.**.**.**.*******.********..*..*** *** ***************.**

iturin A        181 GVMLTHHNLIHNTCAIGTALAIHSKDSFLSWMPLTHDMGLIACHLVPFITGINQNLMPTE 240
bacillomycin L  181 GVMLTHHNLIHNTCAIGTALHVHSKDSFLSWMPLTHDMGLIACHLVPFITGINQNLMPTE 240
bacillomycin D  181 GVMLTHHNLIHNTCAIRNALSIDSKDSFLSWMPLTHDMGLIACHLVPFLVGTNQNLMPTE 240
bacillomycin F  181 GVMLTHHNLIHNTCAIRNALSIDSKDSFLSWMPLTHDMGLIACHLVPFLVGINQNLMPTE 240
mycosubtilin    181 GVMLTHHNLIHNTCAIRNALAIDLKDTLLSWMPLTHDMGLIACHLVPALAGINQNLMPTE 240
mojavensin      181 GVMLTHHNLIHNTRAIRSALALDSKDAFLSWMPLTHDMGLIAFHLAPILVGINQNLMPTE 240
                    *************.**. ** ...**..**************.**.*.. *.********

iturin A        241 LFIRRPILWMKKAHEHKASILSSPNFGYNYFLKFLKN--EPDWDLSHIKVIANGAEPILP 298
bacillomycin L  241 LFIRRPILWMKKAHEHKASILSSPNFGYNYFLKFLKN--EPDWDLSHIKVIANGAEPILP 298
bacillomycin D  241 LFIRRPILWMKKAHEHKCSILSSPNFGYNYFLKFLKN--EPDWDLSHIRVIANGAEPILP 298
bacillomycin F  241 LFIRRPILWMKKAHEHKTSILSSPNFGYNYFLKFLKN--EPDWDLSHIRVIANGAEPILP 298
mycosubtilin    241 LFIRRPILWMKKAHEHKASILSSPNFGYNYFLKFLKDNKSYDWDLSHIRVIANGAEPILP 300
mojavensin      241 LFIRRPILWMKKAHEHKASILSSPNFGYNYFLKFLKD-KSFDWDLSHIRVIANGAEPILP 299
                    *****************.******************.  ..*******.***********

iturin A        299 ELCDEFLKRCAAFNLKRSAILNVYGLAEASVGAAFSKIGKEFVPVYLHRDYLNLGERAVN 358
bacillomycin L  299 ELCDEFLKRCAAFNLKRSAILNVYGLAEASVGAAFSKIGKEFVPVYLHRDYLNLGERAVK 358
bacillomycin D  299 ELCDEFLTRCAAFNMKRSAILNVYGLAEASVGATFSKTGEEFVPVYLHRDHLNLGEKAVK 358
bacillomycin F  299 ELCDEFLKRCATFNMKRSAILNVYGLAEASVGATFSKTGEEFVPVYLHRDHLNLGERAVK 358
mycosubtilin    301 ELCDEFLTRCAAFNMKRSAILNVYGLAEASVGATFSNIGERFVPVYLHRDHLNLGERAVE 360
mojavensin      300 ELCDEFLSRCAVFNMKRSAILNVYGLAEASVGAACSKIGEEFVPVYLHRDHLNLGERAVE 359
                    ******* ***.**.****************** .*..*..*********.*****.**

iturin A        359 VSKEDQNCASFVEVGQPIDYCQLRISDETNERVEDGIIGHIQIKGDNVTQGYYNNPESTE 418
bacillomycin L  359 VSKEDQNCASFVEVGQPIDYCQLRISDETNERVEDGIIGHIHIKGDNVTQGYYNNPESTE 418
bacillomycin D  359 VSKEDQNCASFVEVGQPIDYCQIRISDEANERLEDGIIGHIHIKGDNVTQGYYNNPESTD 418
bacillomycin F  359 VSKEDQNCASFVEVGQPIDYCQIRISNGENERAEDGIIGHIQIKGDNVTQGYYNNPESTD 418
mycosubtilin    361 VSKEDQNCASFVEVGKPIDYCQIRICNEANEGLEDGFIGHIQIKGENVTQGYYNNPESTN 420
mojavensin      360 VSKEDQNCASFVEVGQPIDYCQIRICDEANERLEDGFIGHIQIKGENVTQGYYNNPESTN 419
                    ***************.******.**... **. ***.****.***.*************

iturin A        419 KVLTSDGWVKTGDLGFISESGNLVVTGREKDIIFVNGKNIYPHDIERVAIEMEEVDLGRV 478
bacillomycin L  419 KALTSDGWVKTGDLGFISESGNLVVTGREKDIIFVNGKNIYPHDIERVAIEMEEVDLGRV 478
bacillomycin D  419 RALTSDGWIKTGDLGFISESGNLVVTGREKDIIFVNGKNIYPHDIERVAIELEEVDLGRV 478
bacillomycin F  419 RALTSDGWVKTGDLGFISESGNLVVTGREKDIIFVNGKNIYPHDIERVAIEMEEVDLGRV 478
mycosubtilin    421 RALTPDGWVKTGDLGFIRK-GNLVVTGREKDIIFVNGKNVYPHDIERVAIELEDIDLGRV 479
mojavensin      420 RALTPDGWMKTGDLGFIRE-GNLVVTGREKDIIFVNGKNVYPHDIERVAMEMEGVDLGRV 478
                    ..**.***.********...*******************.*********.*.*..*****

iturin A        479 AACGVYDQKTQSGEIVLFVVYKKSPEKFAPLVKEIKKHLYKRGGWSIKEVLPIRKLPKTT 538
bacillomycin L  479 AACGVYDQKTQSGEIVLFVVYKKSPEKFAPLVKEIKKHLYKRGGWSIKEVLPIRKLPKTT 538
bacillomycin D  479 AACGVYDQNTRSGEIVLFVVFKKSPEKFAPLVKEIKKHLYQRGGWSIKEVLPIRKLPKTT 538
bacillomycin F  479 AACGVYDQKTRSGEIVLFVVFKKSPEKFAPLVKEIKKYLYKRGGWNIKEVLPIRKLPKTT 538
mycosubtilin    480 AACGVYDQETRSREIVLFAVYKKSAEQFAPLVKDIKKHLYQRGGWSIKEILPIRKLPKTT 539
mojavensin      479 AACGIYDQETRSSEIVLFVVFKKSPDQFAPLVKDIKKHLYQRGGWSIKEVLPIRKLPKTT 538
                    ****.*** *.*.*****.* ***...******.***.** ****.***.**********

iturin A        539 SGKVKRYELARQYEAGNFSTESAAINECLESSPETSGQTPIHEIET 584
bacillomycin L  539 SGKVKRYELARQYEAGNFSTESAAINECLESSPETSGQTPIHEIET 584
bacillomycin D  539 SGKVKRYELARQYEAGEFSIESAAINEFLEGSPEKSGQTPIHEIET 584
bacillomycin F  539 SGKVKRYELARQYEAGNFSIESAAINEFLESSPEKSGQTPIHEIET 584
mycosubtilin    540 SGKVKRYELAEQYESGKFALESTKIKEFLEGHSTEPVQTPIHEIET 585
mojavensin      539 SGKVKRYELAEQYESGTFSIESAKIKECLESNPKEPVQTPIHEIET 584
                    **********.***.* *. **..*.* **.... ..*********
```

Supplementary Figure 6. **Amino acid sequence alignment of AL domains.** Asterisks under the amino acids indicate that the amino acids are the same. Points under the amino acids indicate that the amino acids are similar. Amino acid residues mediating the specificity of fatty acids as substrates are shown in bold.

iturin A = AL domain of iturin synthetase derived from *B. subtilis* RB14 (GenBank: BAB69698.1).

bacillomycin L = AL domain of bacillomycin L synthetase derived from *B. velezensis* KACC18228 (GenBank: KSW05788.1).

bacillomycin D = AL domain of bacillomycin D synthetase derived from *B. amyloliquefaciens* CHCC26933 (GenBank: RDY88799.1).

bacillomycin F = AL domain of bacillomycin F synthetase derived from *B. inaquosorum* KCTC13429 (GenBank: AWM17130.1).

mycosubtilin = AL domain of mycosubtilin synthetase derived from *B. subtilis* ATCC6633 (GenBank: QCY17357.1).

mojavensin = AL domain of mojavensin synthetase derived from *B. swezeyi* NRRL B-41282 (GenBank: OMI00188.1).

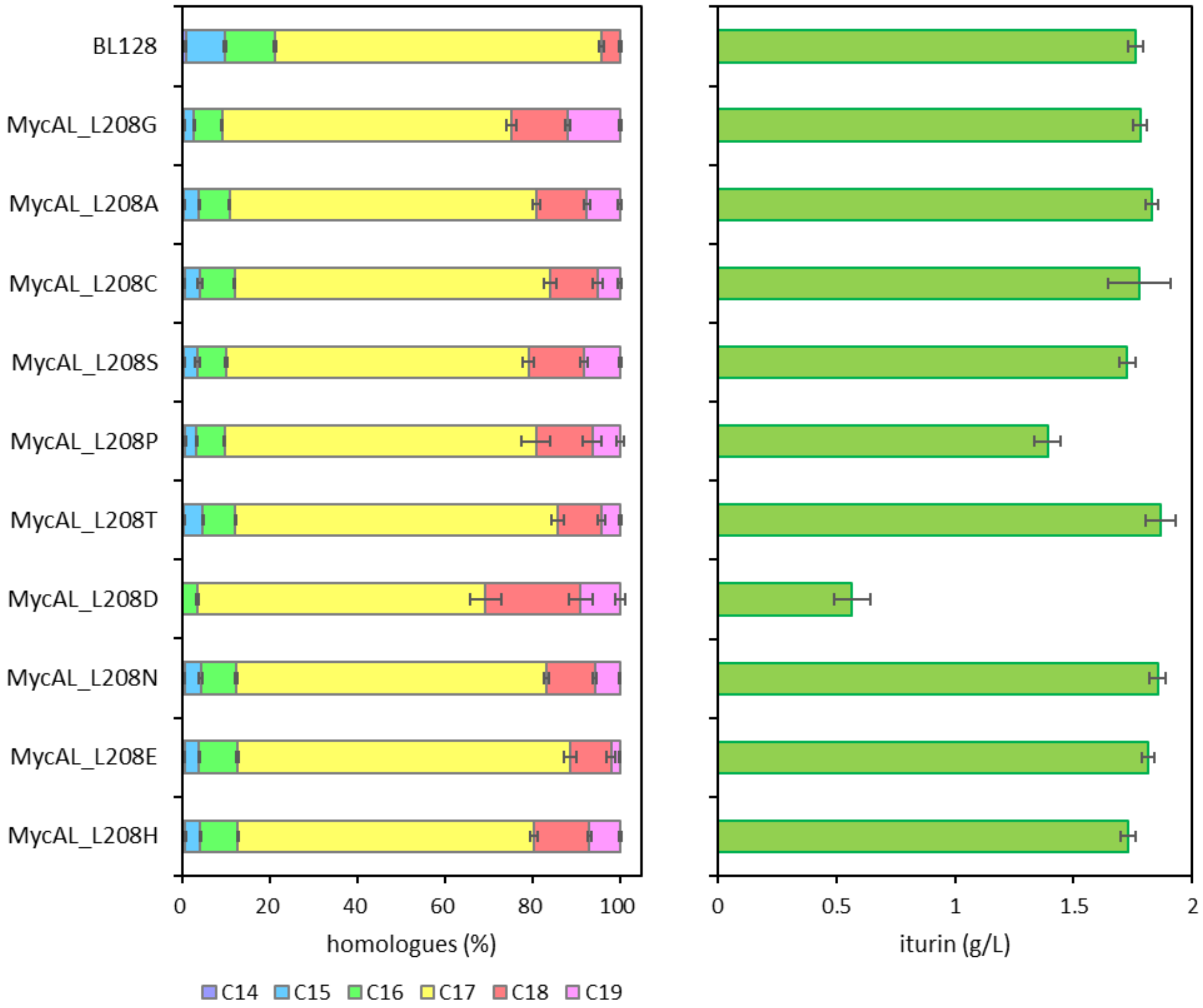

Supplementary Figure 7. Effect of amino acid substitutions in the MycAL on iturin production. The relative abundance and productivity of iturin produced by mutant strains with mutations at L208 in the MycAL. Values and error bars represent the mean and the standard deviation, respectively, of four independent experiments.

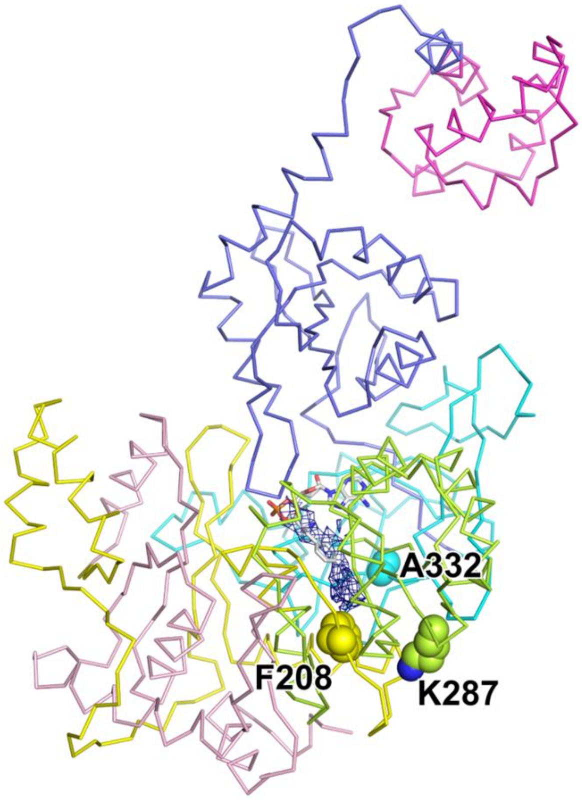

Supplementary Figure 8. **Structure of the iturin synthetase AL domain with the mycosubtilin synthetase ACP domain predicted by ColabFold.** The colours of the ribbon model of the predicted iturin synthetase show the regions used in the experiment for searching amino acid residues affecting substrate specificity. The three amino acid residues mediating the specificity of fatty acids as substrates are represented by a space-filling model. The colours of space-filling models of carbon show the region to which the amino acid residue belongs. The stick model is the phenylalanyl-acyl-AMP observed in the crystal structure of MycG A-PCP (PDB ID: 4R0M) superimposed on the predicted structure. The caged model shows the cavity as the putative binding site for fatty acid as a substrate.

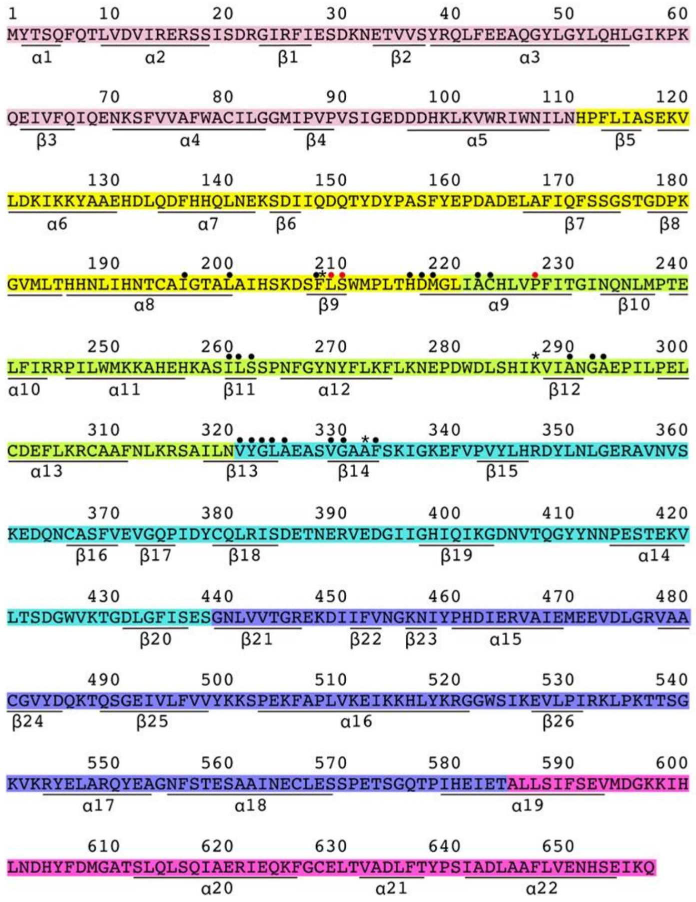

Supplementary Figure 9. **Amino acid sequence of the iturin synthetase AL domain with the mycosubtilin synthetase ACP domain.** The secondary structures obtained from the predicted structure are shown below the sequences. The residues involved in the formation of the substrate-binding pocket are indicated by black circles above the sequence. The three amino acid residues mediating the specificity of fatty acids as substrates are indicated by asterisks above the sequence. The background colour shows the region used in the search for amino acid residues affecting the substrate specificity in Supplementary Fig. 2, and the colouring scheme follows the one for the ribbon model in Supplementary Fig. 8.

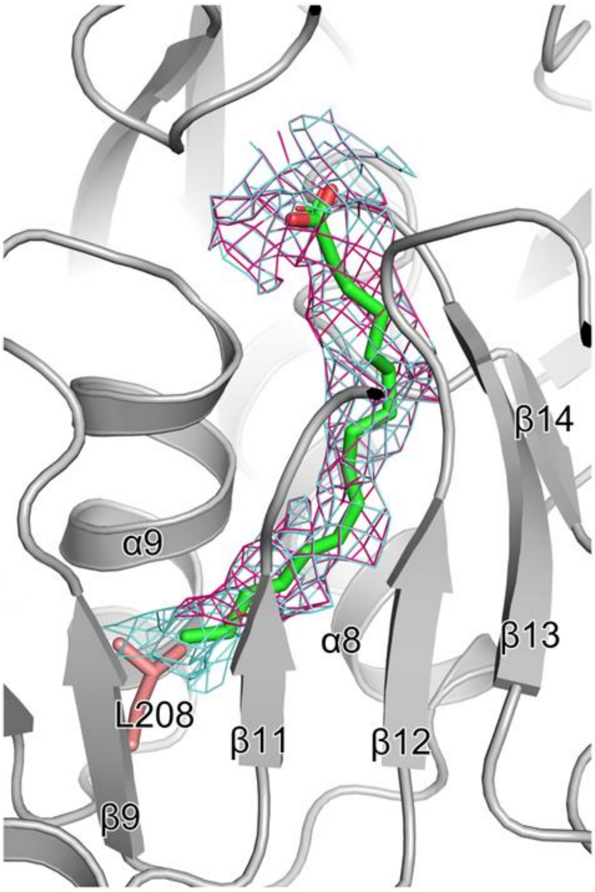

Supplementary Figure 10. **Substrate-binding pockets of native and L208G variant of mycosubtilin synthetase AL domain.** The structural model of native mycosubtilin synthetase AL domain predicted was shown by the grey cartoon model with a pink stick model of Leu208. The grid points of mesh models are the positions of 1 Å away from the nearest atom, and those of native and L208G variant were coloured in red and cyan. The cartoon model with green carbon is a model of palmitic acid with 16 carbon atoms. This structural prediction supported that L208G point mutation made the fatty acid-binding pocket deeper so that the variant accepted fatty acids whose chain length is longer than or equal to C15.

Supplementary Table 1. ***m/z*** **ratio of [M+H]$^+$ ions of the 10 peaks detected by LC-TOF-MS analysis of lipopeptides produced by the F208G mutant strain and the corresponding iturin homologues.**

Peak No.	*m/z*	homologue
1	1043.6	C14
2	1057.6	C15
3	1057.6	C15
4	1071.6	C16
5	1071.6	C16
6	1085.6	C17
7	1085.6	C17
8	1099.6	C18
9	1099.6	C18
10	1113.6	C19
11	1113.6	C19

Supplementary Table 2. **Product ions from the fragmentation of precursor ions listed in Supplementary Table 1.**

Peak No.	*m/z*	AA1	AA2	AA3	AA4	AA5	AA6	AA7	Side Chain
1	1043.553	Asn	Tyr	Asn	Gln	Pro	Asn	Ser	SC(C14)[1]
	915.489	Asn	Tyr	Asn	-	Pro	Asn	Ser	SC(C14)[1]
	801.448	Asn	Tyr	-	-	Pro	Asn	Ser	SC(C14)[1]
	638.386	Asn	-	-	-	Pro	Asn	Ser	SC(C14)[1]
	299.135	-	-	-	-	Pro	Asn	Ser	-
	212.1	-	-	-	-	Pro	Asn	-	-
	184.205	-	-	-	-	-	-	-	SC(C14)[2]
	136.075	-	Tyr[3]	-	-	-	-	-	-
2	1057.572	Asn	Tyr	Asn	Gln	Pro	Asn	Ser	SC(C15)[1]
	929.51	Asn	Tyr	Asn	-	Pro	Asn	Ser	SC(C15)[1]
	815.469	Asn	Tyr	-	-	Pro	Asn	Ser	SC(C15)[1]
	652.404	Asn	-	-	-	Pro	Asn	Ser	SC(C15)[1]
	299.135	-	-	-	-	Pro	Asn	Ser	-
	212.103	-	-	-	-	Pro	Asn	-	-
	198.221	-	-	-	-	-	-	-	SC(C15)[2]
	136.076	-	Tyr[3]	-	-	-	-	-	-
3	1057.571	Asn	Tyr	Asn	Gln	Pro	Asn	Ser	SC(C15)[1]
	929.51	Asn	Tyr	Asn	-	Pro	Asn	Ser	SC(C15)[1]
	815.466	Asn	Tyr	-	-	Pro	Asn	Ser	SC(C15)[1]
	652.403	Asn	-	-	-	Pro	Asn	Ser	SC(C15)[1]
	299.136	-	-	-	-	Pro	Asn	Ser	-
	212.103	-	-	-	-	Pro	Asn	-	-
	198.222	-	-	-	-	-	-	-	SC(C15)[2]
	136.075	-	Tyr[3]	-	-	-	-	-	-
4	1071.59	Asn	Tyr	Asn	Gln	Pro	Asn	Ser	SC(C16)[1]
	943.529	Asn	Tyr	Asn	-	Pro	Asn	Ser	SC(C16)[1]
	829.486	Asn	Tyr	-	-	Pro	Asn	Ser	SC(C16)[1]
	666.422	Asn	-	-	-	Pro	Asn	Ser	SC(C16)[1]
	299.136	-	-	-	-	Pro	Asn	Ser	-
	212.238	-	-	-	-	-	-	-	SC(C16)[2]
	212.103	-	-	-	-	Pro	Asn	-	-
	136.076	-	Tyr[3]	-	-	-	-	-	-

5	1071.587	Asn	Tyr	Asn	Gln	Pro	Asn	Ser	SC(C16)[1)]
	943.527	Asn	Tyr	Asn	-	Pro	Asn	Ser	SC(C16)[1)]
	829.479	Asn	Tyr	-	-	Pro	Asn	Ser	SC(C16)[1)]
	666.419	Asn	-	-	-	Pro	Asn	Ser	SC(C16)[1)]
	299.134	-	-	-	-	Pro	Asn	Ser	-
	212.236	-	-	-	-	-	-	-	SC(C16)[2)]
	212.103	-	-	-	-	Pro	Asn	-	-
	136.075	-	Tyr[3)]	-	-	-	-	-	-
6	1085.605	Asn	Tyr	Asn	Gln	Pro	Asn	Ser	SC(C17)[1)]
	957.542	Asn	Tyr	Asn	-	Pro	Asn	Ser	SC(C17)[1)]
	843.494	Asn	Tyr	-	-	Pro	Asn	Ser	SC(C17)[1)]
	680.435	Asn	-	-	-	Pro	Asn	Ser	SC(C17)[1)]
	299.135	-	-	-	-	Pro	Asn	Ser	-
	226.253	-	-	-	-	-	-	-	SC(C17)[2)]
	212.103	-	-	-	-	Pro	Asn	-	-
	136.075	-	Tyr[3)]	-	-	-	-	-	-
7	1085.604	Asn	Tyr	Asn	Gln	Pro	Asn	Ser	SC(C17)[1)]
	957.543	Asn	Tyr	Asn	-	Pro	Asn	Ser	SC(C17)[1)]
	843.496	Asn	Tyr	-	-	Pro	Asn	Ser	SC(C17)[1)]
	680.435	Asn	-	-	-	Pro	Asn	Ser	SC(C17)[1)]
	299.135	-	-	-	-	Pro	Asn	Ser	-
	226.253	-	-	-	-	-	-	-	SC(C17)[2)]
	212.103	-	-	-	-	Pro	Asn	-	-
	136.075	-	Tyr[3)]	-	-	-	-	-	-
8	1099.619	Asn	Tyr	Asn	Gln	Pro	Asn	Ser	SC(C18)[1)]
	971.559	Asn	Tyr	Asn	-	Pro	Asn	Ser	SC(C18)[1)]
	857.515	Asn	Tyr	-	-	Pro	Asn	Ser	SC(C18)[1)]
	694.451	Asn	-	-	-	Pro	Asn	Ser	SC(C18)[1)]
	299.135	-	-	-	-	Pro	Asn	Ser	-
	240.269	-	-	-	-	-	-	-	SC(C18)[2)]
	212.103	-	-	-	-	Pro	Asn	-	-
	136.075	-	Tyr[3)]	-	-	-	-	-	-
9	1099.619	Asn	Tyr	Asn	Gln	Pro	Asn	Ser	SC(C18)[1)]
	971.561	Asn	Tyr	Asn	-	Pro	Asn	Ser	SC(C18)[1)]
	857.515	Asn	Tyr	-	-	Pro	Asn	Ser	SC(C18)[1)]
	694.452	Asn	-	-	-	Pro	Asn	Ser	SC(C18)[1)]
	580.405	-	-	-	-	Pro	Asn	Ser	SC(C18)
	299.136	-	-	-	-	Pro	Asn	Ser	-
	240.269	-	-	-	-	-	-	-	SC(C18)[2)]
	212.104	-	-	-	-	Pro	Asn	-	-
	136.076	-	Tyr[3)]	-	-	-	-	-	-

10	1113.636	Asn	Tyr	Asn	Gln	Pro	Asn	Ser	SC(C19)[1)]
	985.576	Asn	Tyr	Asn	-	Pro	Asn	Ser	SC(C19)[1)]
	871.531	Asn	Tyr	-	-	Pro	Asn	Ser	SC(C19)[1)]
	708.469	Asn	-	-	-	Pro	Asn	Ser	SC(C19)[1)]
	594.423	-	-	-	-	Pro	Asn	Ser	SC(C19)
	299.136	-	-	-	-	Pro	Asn	Ser	-
	254.286	-	-	-	-	-	-	-	SC(C19)[2)]
	212.103	-	-	-	-	Pro	Asn	-	-
	136.075	-	Tyr[3)]	-	-	-	-	-	-
11	1113.633	Asn	Tyr	Asn	Gln	Pro	Asn	Ser	SC(C19)[1)]
	985.573	Asn	Tyr	Asn	-	Pro	Asn	Ser	SC(C19)[1)]
	871.528	Asn	Tyr	-	-	Pro	Asn	Ser	SC(C19)[1)]
	708.466	Asn	-	-	-	Pro	Asn	Ser	SC(C19)[1)]
	594.422	-	-	-	-	Pro	Asn	Ser	SC(C19)
	299.134	-	-	-	-	Pro	Asn	Ser	-
	254.284	-	-	-	-	-	-	-	SC(C19)[2)]
	212.102	-	-	-	-	Pro	Asn	-	-
	136.075	-	Tyr[3)]	-	-	-	-	-	-

1) N- and C-termini were assumed to be connected by an amide bond.

2) A part of the fatty acid side chain [NH-CH-$(CH_2)_{n-4}$-CH_3] was detected.

3) It was detected as an iminium ion (an ion from which carbonyl was removed).

Supplementary Table 3. **Relative abundance of each fatty acid.**

Fatty acid	Relative abundance (%)	
	Precipitates of the culture broth of the F208G mutant strain	Precipitates of a production medium
iso-C14:0	0.2 ± 0.02	N. D.
iso-C15:0	3.4 ± 0.2	N. D.
anteiso-C15:0	11.6 ± 0.6	N. D.
iso-C16:0	1.2 ± 0.09	N. D.
n-C16:0	7.7 ± 0.2	9.9 ± 1.8
iso-C17:0	3.8 ± 0.1	N. D.
anteiso-C17:0	5.9 ± 0.1	N. D.
n-C18:1, C18:2, C18:3	64.1 ± 1.4	87.0 ± 2.2
n-C18:0	2.1 ± 0.06	3.1 ± 0.4

* N. D. = Not Detected
