# Supplementary material for: Engineering of acyl ligase domain in non-ribosomal peptide synthetases to change fatty acid moieties of lipopeptides
Source: Commun Chem. 2025 Jan 21;8:17. doi: 10.1038/s42004-024-01379-w (PMC11751314; doi:10.1038/s42004-024-01379-w)
Supplement: Supplementary file 3 — Description of Additional Supplementary Files [file 42004_2024_1379_MOESM3_ESM.pdf]

Description of Additional Supplementary Files

File name: Supplementary Data 1

Description: Primers used in this study
